# The role of sleep dysfunction in the occurrence of delusions and hallucinations: A systematic review

**DOI:** 10.1016/j.cpr.2015.09.001

**Published:** 2015-12

**Authors:** Sarah Reeve, Bryony Sheaves, Daniel Freeman

**Affiliations:** Department of Psychiatry, University of Oxford, UK

**Keywords:** Sleep, Insomnia, Psychosis, Delusions, Hallucinations, Schizophrenia

## Abstract

**Background:**

Sleep dysfunction is extremely common in patients with schizophrenia. Recent research indicates that sleep dysfunction may contribute to psychotic experiences such as delusions and hallucinations.

**Objectives:**

The review aims to evaluate the evidence for a relationship between sleep dysfunction and individual psychotic experiences, make links between the theoretical understanding of each, and highlight areas for future research.

**Method:**

A systematic search was conducted to identify studies investigating sleep and psychotic experiences across clinical and non-clinical populations.

**Results:**

66 papers were identified. This literature robustly supports the co-occurrence of sleep dysfunction and psychotic experiences, particularly insomnia with paranoia. Sleep dysfunction predicting subsequent psychotic experiences receives support from epidemiological surveys, research on the transition to psychosis, and relapse studies. There is also evidence that reducing sleep elicits psychotic experiences in non-clinical individuals, and that improving sleep in individuals with psychosis may lessen psychotic experiences. Anxiety and depression consistently arise as (partial) mediators of the sleep and psychosis relationship.

**Conclusion:**

Studies are needed that: determine the types of sleep dysfunction linked to individual psychotic experiences; establish a causal connection between sleep and psychotic experiences; and assess treatments for sleep dysfunction in patients with non-affective psychotic disorders such as schizophrenia.

## Highlights

• The review assesses the link between sleep dysfunction and psychotic experiences, particularly delusions and hallucinations.
• A systematic review is undertaken of 66 papers that assessed sleep and individual psychotic experiences.
• Sleep dysfunction and psychosis clearly co-occur and sleep dysfunction may predict later psychotic experiences.
• The effect of improving sleep on psychotic experiences remains to be tested in an adequately powered trial.
• The mechanisms linking sleep dysfunction to psychotic experiences require testing.

## 1 Introduction

Psychotic disorders such as schizophrenia have been associated with sleep dysfunction since first clinically described. In case study descriptions Kraepelin (1919) frequently refers to disturbed sleep and suggests that in treatment “Rest in bed, supervision, care for sleep and food, are here the most important requisites” (p. 279). Bleuler (1950) also noted that sleep disturbances were common in psychosis: “Sleep is habitually disturbed... Many patients do not want to sleep because they want to know what goes on during the night, or because they fear some violence to themselves while asleep” (p. 59). Patient accounts similarly show awareness of an interaction between their psychotic experiences and their sleep: “They [the voices] keep me from going to sleep”; “the more tired I am the worse they get” (Waite et al., 2015). However, it is only in the last few years that a research agenda has emerged concerning sleep dysfunction as a putative contributory causal factor and therapeutic target in the occurrence of psychotic experiences (Freeman et al., 2009, Freeman et al., 2015, Harvey and Murray, 2011, Wulff et al., 2010).

Outside of the clinical realm a literature exists showing the effects of sleep deprivation on individuals in the general population. Small studies carried out in the 1950s and 1960s reported that sleep deprivation in otherwise healthy individuals leads to emergence of psychotic-like experiences, such as hallucinations, with the frequency and severity of these experiences increasing with time spent awake (Bliss et al., 1959, Brauchi and West, 1959, Katz and Landis, 1935, Luby et al., 1960, Pasnau et al., 1968, Ross, 1965, West and Janszen, 1962). Illustrative descriptions from these studies include: “He saw a fine smoke begin to rise from the floor….as he stared at the floor more closely, fine jets of water appeared to be rising” (Bliss et al., 1959) and: “During the course of the vigil Z [attributed] persecutory intent to one of the experimenters. He became more and more certain that this experimenter was personally interested in making life disagreeable for him” (Katz & Landis, 1935) Intriguingly, one experimental study found that 100 h of wakefulness was associated with a resurgence or exaggeration of psychotic symptoms in a small sample (n = 6) of inpatients with schizophrenia (Koranyi & Lehmann, 1960).

In this review we revisit the link between disrupted sleep and individual psychotic experiences, in particular delusions and hallucinations. We set out to answer the question of whether psychotic experiences and sleep dysfunction are related, and if so, how. After reviewing the existing research addressing these questions we will then integrate its findings into the theoretical literature of psychosis and sleep, in the process highlighting priorities for future research.

### 1.1 Psychotic experiences

This review is intended to cover the relationship between sleep dysfunction and the main positive psychotic experiences — hallucinations and delusions. The empirical research indicates that non-affective psychotic diagnoses such as schizophrenia actually contain multiple independent experiences, including paranoia, grandiosity, and hallucinations (Peralta and Cuesta, 1999, Ronald et al., 2014, Vazquez-Barquero et al., 1996, ???). Heritabilities of each type of psychotic experience have been found to vary (Zavos et al., 2014).

Whilst the individual psychotic experiences are typically studied in psychotic disorders such as schizophrenia, they are also common in the general population, with the prevalence of hallucinations and delusions estimated from epidemiological studies to be in the region of 7–11% (Linscott & van Os, 2013). In clinical groups, the association of the individual psychotic experiences is inflated, due to Berkson's bias, whereby individuals with multiple problems are more likely to come into contact with clinical services (Bak et al., 2005, Maric et al., 2004). The individual psychotic experiences exist on spectra of severity in the general population; in other words, like emotional disorders (Plomin, Haworth, & Davis, 2009), they exist as quantitative traits in the general population, with clinical populations experiencing the severe end of the continua. A focus on individual psychotic experiences has allowed development of detailed theoretical models, for example for paranoia (Freeman, Garety, Kuipers, Fowler, & Bebbington, 2002) accompanied by corresponding innovations in treatment (Freeman & Garety, 2014).

The evidence is threefold supporting the idea that non-clinical and clinical psychotic experiences exist on the same continuum: in both groups they are associated with similar environmental (e.g. trauma, cannabis use) and psychological factors (e.g. anomalous experiences, worry) (Freeman et al., 2010b, Johns and van Os, 2001); milder psychotic experiences are more common in families of individuals with psychotic disorders than families without such a history (e.g. van Os, Linscott, Myin-Germeys, Delespaul, & Krabbendam, 2009); and adolescents who report psychotic experiences are at greater risk of developing psychotic disorders in adulthood (e.g. Fisher, Caspi, & Poulton, 2013).

Therefore, in contrast with existing reviews linking sleep and schizophrenia (Benson, 2006, Benson, 2008, Cohrs, 2008, Lunsford-Avery and Mittal, 2013, Monti and Monti, 2004, Pritchett et al., 2012, Waters and Manoach, 2012, Zanini et al., 2013), this article will particularly focus on research that investigates the association of sleep dysfunction with individual psychotic experiences, with attention also paid to studies that jointly assess delusions and hallucinations as positive symptoms. We will exclude schizotypy studies, unless they separate out individual psychotic experiences.

### 1.2 Sleep and sleep dysfunction

The regulation of sleep can be conceptualised in a two process model (Borbély, 1982) comprising a circadian component (intrinsic synchronisation of body functions to the light–dark cycle) and a homeostatic component, commonly referred to as ‘sleep pressure’ or need for sleep, which accumulates during wakefulness and is dissipated by sleep. Sleep itself can be divided into rapid eye movement (REM) and non-rapid eye movement (NREM) sleep stages. NREM sleep can further be divided into stages 1, 2, 3 and 4 in order of increasingly deeper sleep. Stages 3 and 4 are characterised by the presence of slow, high amplitude waves of neural activity and are often referred to jointly as slow wave sleep (SWS) or delta sleep. A typical sleep phase passes through NREM stages 1 to 4 before moving to a REM sleep stage. This cycle takes around 90 min, and repeats throughout the night, with an increasing predominance of REM sleep in the latter half of the night (Brown, Basheer, McKenna, Strecker, & McCarley, 2012).

Using polysomnography (PSG — see Table 1a) these stages can be identified, providing several objective measures of sleep. Another objective measure of sleep can be gained from actigraphy. This technique requires participants to wear a wrist watch style device which monitors their movement and light exposure, usually over several weeks. This can then allow interpolation of sleep–wake and circadian function (when combined with melatonin sequencing). Key sleep parameters relevant to this review are listed in Table 1b.

Sleep disorders comprise a broad range of disturbances (see Table 2). All of these disorders are associated with a subjective complaint of dissatisfaction in quality, timing, or amount of sleep, with diagnosis requiring daytime distress and/or impairment. Definitive diagnosis of several sleep disorders (for example sleep apnoea) requires polysomnographic assessment, although this is not time or cost-efficient to carry out on a large scale. Therefore, many studies investigating sleep dysfunction rely on self-report of dissatisfaction with sleep quality, timing, or amount of sleep. These measures may then be considered as indexing symptoms of a sleep disorder such as insomnia (or other sleep disorders with appropriate additional symptom reports, e.g. nightmares) or be simply taken as a measure of lack of satisfaction with sleep.

In this review, unless otherwise specified, sleep dysfunction will be used as a broad term to encompass abnormalities in objective sleep variables (Table 1b), sleep disorders or symptoms (Table 2), and also subjective dissatisfaction with sleep in the absence of a sleep disorder diagnosis.

### 1.3 Sleep dysfunction in schizophrenia

Schizophrenia is the most common non-affective psychotic disorder, and a diagnosis long anecdotally linked with sleep dysfunction. Therefore when reviewing the link between sleep dysfunction and psychotic experiences, it is worthwhile to first consider what has been learned about the relationship between sleep dysfunction and schizophrenia. However, there is a great deal of variability reported in the literature. In part this can be explained by the heterogeneity of groups studied, for example, in symptoms, age, medication status, and also by the small sample sizes imposed by the demands of PSG. For example, most of the sleep variables listed in Table1b have been reported to be altered in patients with schizophrenia (Monti & Monti, 2004). Yet a meta-analysis by Chouinard, Poulin, Stip, and Godbout (2004) found that medication-free patients with schizophrenia only exhibited increased sleep latency, decreased total sleep time, and decreased sleep efficiency. Furthermore, treatment naïve patients were found to exhibit a different set of sleep characteristics in comparison to the previously treated patients. This highlights the complex interaction of sleep dysfunction with symptom and medication status. Various circadian rhythm alterations such as melatonin phase and sleep/wake pattern irregularity have also been widely reported among patients with schizophrenia, and have previously been proposed to have etiological and therefore therapeutic relevance to the disorder (Anderson and Maes, 2012, Monti et al., 2013). Yet again, the interaction of circadian processes with antipsychotic medication and the wide range of dysfunction recorded prevent more detailed conclusions. Overall, it is clear that many aspects of sleep architecture are altered in individuals with schizophrenia, although often the interpretation of a particular difference is challenging due to the lack of consensus linking particular aspects of sleep architecture (e.g. decreased stage 2 sleep) to daytime functioning (Wilson & Argyropoulos, 2012).

Where data exist, they indicate that prevalence rates of sleep disorders are elevated in schizophrenia. The majority of research has focused on insomnia, with estimates for the prevalence of clinically significant insomnia among individuals with a diagnosis of schizophrenia varying from 36 to 80%, depending on study recruitment criteria and definition of insomnia used (Freeman et al., 2009, Palmese et al., 2011, Xiang et al., 2009). Regardless, even the lowest estimates support the conclusion that rates of insomnia are elevated among patients with schizophrenia as compared to the general population (see Table 2). Rates of sleep apnoea also appear elevated in individuals with schizophrenia — around 15% of patients have been found to exhibit clinically significant symptoms (Sharafkhaneh, Giray, Richardson, Young, & Hirshkowitz, 2005). The link between sleep apnoea and schizophrenia is likely to be partly mediated by the increased BMI in patients with schizophrenia, which is itself contributed to by long term use of antipsychotics and lifestyle factors (Winkelman, 2001). However there is a possible direct contribution of particular antipsychotic medications via their effect on muscle tone in the upper airway (Kalucy, Grunstein, Lambert, & Glozier, 2013). Symptoms of hypersomnia such as excessive daytime sleepiness and extended nocturnal sleep period are also commonly observed in patients with schizophrenia, and are again likely to be partly influenced by medication (Hawley, 2006, Hawley et al., 2010). For instance, one initial empirical study suggested that daytime sleep propensity is lower in patients with schizophrenia than in the non-clinical population, yet the researchers also found that antipsychotic medication increased sleep propensity and subjective complaints of sleepiness (Kluge et al., 2012). No aggregated figure for parasomnia prevalence is available for patients with schizophrenia, although there is recent evidence for an increased rate of nightmares among individuals with psychotic disorders (Michels et al., 2014, Sheaves et al., 2015a).

Despite the apparent high prevalence of sleep disorders in patients with schizophrenia, and improving sleep being among patients' highest priorities for treatment (Auslander and Jeste, 2002, Waite et al., 2015), sleep disorders in schizophrenia are rarely addressed directly. Clinicians and researchers have traditionally described sleep difficulties in schizophrenia as a secondary symptom, or simply ascribed their frequent presentation to the effects of antipsychotic medication or social isolation (Wilson & Argyropoulos, 2012). Yet many sleep disorders have existing effective treatments, which are beginning to be validated in psychiatric groups (e.g. Freeman et al., 2015, Myers et al., 2011). This new approach coincides with the recent change in conceptualisation of sleep disorders in diagnostic manuals, with guidelines now in place stating that clinically significant sleep disorders should be considered as a comorbid diagnosis and receive independent clinical attention accordingly, regardless of the presence of other mental or medical conditions (AASS, 2014, APA, 2013).

### 1.4 The current review

This review will restrict itself to examining the association between sleep dysfunction and individual psychotic experiences, specifically focusing on delusions and hallucinations, across clinical and non-clinical populations. We sought to address the following questions:

1. Do sleep dysfunction and psychotic experiences co-occur?
2. Does sleep dysfunction predict onset or persistence of individual psychotic experiences? (or vice versa)
3. Does a change in sleep influence psychotic experiences? (or vice versa)

In the course of this investigation we will also seek to identify factors found to mediate the relationship between sleep and psychotic experiences.

## 2 Method

A search was carried out on PubMed for English language papers published in peer-reviewed journals containing the following terms: (Sleep OR Insomnia OR Dream* OR Nightmare*) AND (Delus* OR Hallucinat* OR Psychosis OR Psychotic OR Schizophren*), published from 1980 until the present day.

### 2.1 Inclusion criteria

Papers were required to have reported all of the below:

1. Explicit measures of psychotic experiences or positive symptoms;
2. Objective or subjective measure of sleep dysfunction;
3. Analysis testing a relationship between these two factors.

### 2.2 Exclusion criteria

Papers fulfilling any of the below criteria were excluded:

1. Single case studies
2. Relating primarily to:
  a. Dementias or other neurological conditions
  b. Bipolar disorder or other affective psychoses (including post-partum psychosis)
  c. Schizotypy or dissociation
  d. Hypnogogic or hypnopompic hallucinations
  e. Narcolepsy

**Fig. 1:**
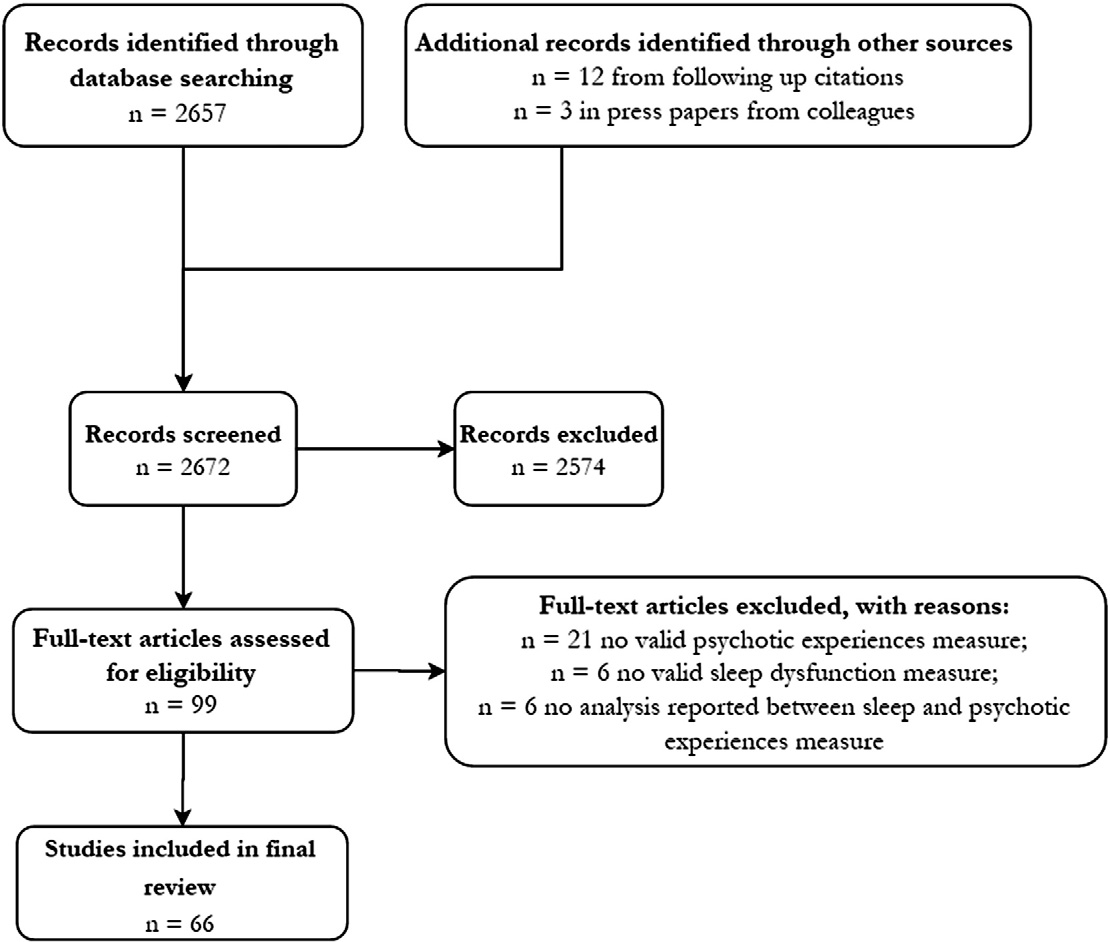
PRISMA Cohort Diagram.

This search revealed 2657 papers (July 2015). Titles and abstracts were scanned, and if appropriate the whole paper, in order to ascertain whether each item fulfilled the inclusion and exclusion criteria. The reference lists of relevant papers were also scanned for further citations. Three papers from within the research group which at the time of the search were currently in revision or in press prior to publication were also included. This yielded a total of 66 papers, which for the purposes of discussion have been divided into investigations on non-clinical and clinical groups. See Fig. 1 for a PRISMA flow diagram of the systematic review process.

## 3 Results

### 3.1 General population

Eighteen papers investigating the relationship between sleep dysfunction and psychotic experiences in the general population were found (see Table 3). The majority of studies utilise subjective reports of either insomnia symptoms (8 papers) or other sleep disturbances such as nightmares (7 papers), while the remaining three papers focus on sleep deprivation.

#### 3.1.1 Insomnia and psychotic experiences

Of the individual psychotic experiences, the relationship between paranoia and insomnia has received the most attention in the literature. Evidence from multiple sources suggests that insomnia and paranoia co-occur. For example, in national epidemiological surveys, a diagnosis of insomnia was associated with odds ratios (OR) of 1.78–2.54 for reporting concomitant paranoia (Freeman et al., 2011), with chronic insomnia linked to a 5 times increased likelihood of endorsing the statement ‘a group of people are plotting to cause you serious harm or injury’ (Freeman, Brugha, et al., 2010). This relationship was partially mediated by depression and anxiety symptoms, and to a more limited extent by cannabis use, although an independent contribution of insomnia symptoms to paranoia remained even after controlling for these factors (Freeman et al., 2009, Freeman et al., 2011).

In addition to the co-occurrence of insomnia with paranoia, longitudinal evidence indicates that insomnia predicts future paranoia. Insomnia was found to predict new paranoid thinking (OR 1.49–1.84) and persistence of existing paranoid thinking (OR = 1.2) at an 18 month follow up of an epidemiological population survey (Freeman et al., 2012). In a smaller study, still using dimensional measures of insomnia and paranoia, insomnia was a significant predictor of paranoia and PTSD symptoms over 6 months (Freeman et al., 2013).

Insomnia has also been found to co-occur with and predict new hallucinatory experiences in the same general population samples (whilst controlling for both affective symptoms and paranoia) (Sheaves et al., submitted). A cross sectional analysis found that insomnia symptoms were associated with a two to four times increased likelihood of endorsing hallucination items relating to hearing or seeing things that others couldn't, or hearing voices saying words or sentences. Longitudinal analysis further revealed that insomnia symptoms as baseline predicted new hallucinatory experiences at 18 month follow up.

More broadly, a large WHO study combining data from 56 countries (n = 267,692) corroborates a cross-sectional relationship between sleep problems (indexed by a single self-report questionnaire item based on insomnia criteria) and several psychotic experiences. Self-reported sleep disruption significantly increased the likelihood of concomitant psychotic experiences (OR = 2.26–2.84 across different psychotic experiences), even after controlling for anxiety and depression (remaining ORs of 1.54–1.68) (Koyanagi & Stickley, 2015).

A recent cross-sectional study examined the genetic and environmental influences on sleep dysfunction and six individual psychotic experiences (Taylor, Gregory, Freeman, & Ronald, 2015). Five thousand 16-year old twin pairs completed self-report assessments for insomnia symptoms, subjective sleep quality, paranoia, hallucinations, cognitive disorganisation, grandiosity, anhedonia, and (parent rated) negative symptoms. The latter three factors were found to only have mild covariance with the sleep measures, but paranoia, hallucinations and cognitive disorganisation all demonstrated a strong association with the sleep dysfunction variables. Further analysis utilising modelling of genetic, environmental, and non-shared environment influences on the sleep and psychotic experience phenotypes demonstrated an aetiological overlap (i.e. shared genetic and environmental mechanisms). These relationships reduced, but again remained significant, after controlling for negative affect. Overall there is strong evidence for insomnia co-occurring with a range of psychotic experiences, and some indications of insomnia predicting later psychotic experiences (limited to paranoia and hallucinations at this point). It should be noted that none of these longitudinal studies had the statistical power to test the reverse association (i.e. psychotic experiences predicting later sleep dysfunction). Furthermore, no studies have attempted to reduce insomnia in a non-clinical population to test the effect on psychotic-like experiences. In terms of mediating factors for the relationship between insomnia and psychotic experiences, there is evidence of partial mediation by depression and anxiety.

#### 3.1.2 Other sleep disorders and psychotic experiences

A co-occurrence between psychotic experiences and sleep disorders other than insomnia is also supported by the literature. A number of sleep disorder symptoms (including excessive daytime sleepiness, cataplexy, and excessive snoring) were found to predict psychotic like experiences in adolescent samples (Lee et al., 2012, Oshima et al., 2010). In the former study this relationship reduced but remained significant after controlling for depressive symptoms.

The likelihood of reporting psychotic experiences in another young sample (aged 11–12) increased with the number of reported sleep problems, including nightmares and daytime fatigue (Jeppesen et al., 2014). Distress associated with nightmares in a student sample was associated with increased paranoia, anxiety, and depression (as well as poorer occupational functioning) (Levin & Fireman, 2002). Findings from the ALSPAC longitudinal cohort have also indicated that parasomnias during childhood (nightmares and night terrors) predict psychotic experiences at age 12 (Fisher et al., 2014), and that nightmares at age 12 predict psychotic experiences at 18 (Thompson et al., 2015) even after adjusting for mood and psychotic experiences at age 12.

Altogether, these studies suggest that the relationship between sleep dysfunction and psychotic experiences is not limited to insomnia, but extends to other sleep disorders, and that these disorders co-occur with and may predict psychotic experiences. However, these findings are primarily based upon adolescent samples, and there is evidence that some sleep disorders may be more common in this group than in the adult population (e.g. parasomnias: Laberge, Tremblay, Vitaro, & Montplaisir, 2000). Therefore further investigation is needed to explore whether these links between parasomnias and excessive daytime sleepiness and psychotic experiences are also present in the adult population.

#### 3.1.3 Sleep deprivation

Three studies have manipulated sleep via sleep deprivation to investigate its impact on psychotic-like experiences.

Kahn-Greene, Killgore, Kamimori, Balkin, and Killgore (2007) deprived 25 non-clinical volunteers of sleep for 56 h, administering the Personality Assessment Inventory (PAI; Morey, 1991) at baseline and after sleep deprivation. Following sleep deprivation, increases were observed in the anxiety, depression and paranoia factors, but not in ‘manic related symptoms’ or ‘schizophrenia symptoms’ factors of the PAI. Yet with the exception of paranoia the scale dimensions do not appear to differentiate positive psychotic experiences from negative symptoms or affective symptoms, therefore it is difficult to interpret these results with regard to particular psychotic experiences.

In the second sleep deprivation study a student sample underwent a night of sleep deprivation or normal sleep in two counterbalanced sessions under laboratory conditions (Petrovsky et al., 2014). The psychotomimetic states inventory (PSI; Mason, Morgan, Stefanovic, & Curran, 2008) – a scale previously utilised for self-report of the effects of cannabis and ketamine on non-clinical volunteers in research contexts – was administered in the evening and morning for both conditions. This experiment found that the sleep deprivation condition resulted in significantly higher ratings in several PSI subscales (perceptual distortions, cognitive disorganisation, and anhedonia) but not others (mania, paranoia or delusional thinking). No data on mood changes were collected. Given the robust link between insomnia and paranoia discussed previously (Freeman et al., 2010a, Freeman et al., 2009, Freeman et al., 2011, Freeman et al., 2012), it is intriguing that no effects of sleep deprivation on paranoia were reported in this study. One potential rationale could be that the 8 item PSI subscale for paranoia includes several items which are less specific to persecutory ideation (e.g. “you feel that you deserve to be punished”, “you feel that no-one understands you”) than the questionnaire items used in the paranoia-focused studies e.g. the Green et al. Paranoid Thoughts Scale (Green et al., 2008).

The last sleep deprivation study identified by the review took a somewhat different approach in utilising natural conditions of sleep deprivation — in this case by competitors taking part in a 168 km ultramarathon event (Hurdiel et al., 2014). Wrist actigraphy was used to track volunteer competitors in this event (involving an average of 46 h 38 min of wakefulness for this group), and on completion of the event a short questionnaire was filled out including an open-ended item asking about experiences of sleep deprivation (including hallucinations). Four of the seventeen volunteers reported experiencing hallucinations during the event.

These studies do indicate that experimentally reducing sleep increases psychotic-like experiences, although the evidence is inconsistent with regard to particular psychotic experiences. Besides the issues already highlighted in the questionnaires employed, other limitations are that all of the above studies have used small samples, and none includes analyses for mediating factors. In the case of the last study, there was also no baseline measurement or control group.

### 3.2 Clinical populations

Table 4, Table 5, Table 6, Table 7a, Table 7b, Table 8 contain the forty-nine clinical studies retrieved by the search terms. Research in these clinical groups is complicated by the heterogeneity of individuals with psychosis, such as differing medication status, age, and current symptom presentation.

Furthermore, many of the studies in clinical populations poorly differentiate between individual psychotic experiences, both in recruitment and in the measures used. Over three quarters of studies retrieved were carried out with a group recruited for a diagnosis of schizophrenia or another psychotic disorder (41 papers), regardless of symptom presentation. Other participants were recruited based on broadly defined psychotic symptoms (5 papers). Another four studies utilise differing criteria (based on a mixture of positive psychotic and other symptoms) for selecting a sample at high risk of psychosis. Only 3 studies have addressed individual psychotic experiences in relation to sleep, and these predominantly focus on paranoia and persecutory delusions.

#### 3.2.1 Polysomnography

Polysomnographic methods have been widely applied in this clinical population (Table 4). All of the studies are cross-sectional designs, using small samples, often seeking to associate clinical severity or symptom profiles with particular differences in sleep architecture.

The most consistently reported findings related to positive psychotic symptoms are REM sleep alterations. Positive symptoms have been found to be associated with a lowered REM onset latency in unmedicated and medication naive patients (Lauer et al., 1997, Poulin et al., 2003, Tandon et al., 1992, Yang and Winkelman, 2006) although there are inconsistent results, mostly from studies with smaller sample sizes (Ganguli, 1987, Tandon et al., 1989, Taylor et al., 1991b). Similarly, reduced REM onset latency and an increased percentage of time in REM sleep were correlated with positive symptom severity (Sarkar, Katshu, Nizamie, & Praharaj, 2010). Another sleep EEG study found that positive symptom scores partially explained the variation (63%) in high frequency EEG activity during REM sleep (Tekell et al., 2005). An association between increased positive psychotic symptoms and reduced REM density has also been reported (Rotenberg et al., 1997, Yang and Winkelman, 2006).

The interpretation of these REM sleep abnormalities is opaque, as the function of REM sleep and the impacts of alterations to it are still the subject of debate (e.g. Wamsley, 2014). Regardless, these changes do at the very least point to a disruption in neural regulation of sleep in individuals with psychosis, which could have an impact throughout the sleep period. For example, a reduced REM sleep onset latency may mean that less time is spent in slow wave sleep prior to first REM epoch.

In contrast to REM sleep, alterations in slow wave sleep have been more traditionally associated with negative symptoms e.g. (Kajimura et al., 1996, Keshavan et al., 1995, Keshavan et al., 1998). Yet three recent polysomnographic studies have reported that a reduced number and amplitude of spindles recorded during slow wave sleep is associated with greater positive symptom severity (Ferrarelli et al., 2010, Sarkar et al., 2010, Wamsley et al., 2012) — although in one case a reverse association (increased sleep spindle amplitude with increased positive symptom score) was reported (Manoach et al., 2014).

However, as all of these polysomnographic studies are cross-sectional they are unable to provide any conclusions as to whether the REM sleep changes occur prior to psychosis or predict other clinical variables in the longer term. Furthermore, in all of these studies clinical participants were not recruited on the basis of particular psychotic experiences, and the measures used amalgamate multiple psychotic experiences, meaning that it is not possible to speculate on whether particular psychotic experiences have differing associations with the sleep architecture changes described.

#### 3.2.2 Actigraphy and circadian rhythm

Several recent studies have used actigraphy to assess sleep wake patterns and circadian dysfunction in psychosis, with the aim of linking circadian profiles to clinical features (Table 5).

One longitudinal study has indicated that sleep–wake abnormalities detected by actigraphy predict psychotic symptoms at one year follow up in a high risk youth sample (Lunsford-Avery, LeBourgeois, Gupta, & Mittal, 2015). Another smaller longitudinal study (clinical group n = 6) found that daily variations in sleep duration were associated with positive symptoms, partially mediated by mood changes, in outpatients with schizophrenia (Waters et al., 2011). Furthermore, the changes in mood for the clinical group partly explained the association between sleep and positive symptom scores. A cross sectional study with a large sample of outpatients with schizophrenia (n = 75) identified a relationship between reduced activity and positive symptom severity (Wichniak et al., 2011). On the other hand, two other cross-sectional studies utilising melatonin profiling as well as actigraphy (Bromundt et al., 2011, Wulff et al., 2012) did not find any significant association between positive symptom scores and sleep–wake or circadian phase abnormalities. Another report from an actigraphic monitoring study on older patients with schizophrenia found no associations between any clinical variables (including positive symptoms) and activity levels (Martin et al., 2001).

From these initial findings, it seems likely that circadian dysfunction plays a part in the observed sleep disruption in individuals with psychosis. However, further studies utilising circadian profiling to link particular characteristics (e.g. advanced phase, delayed phase, free running) with symptomatology are a clear next step. The high diversity of sleep and circadian phenotypes reported in these initial small samples indicates that much larger studies will be required to identify if there are any particular associations by psychotic experience type.

#### 3.2.3 Sleep and clinical course of psychosis

Several different types of study have linked sleep with the clinical course of psychosis, including investigations of individuals at risk of transition to first episode psychosis, retrospective reports following first psychotic episode, and quality of life in patients with non-affective psychosis (Table 6).

An intriguing subset of studies have investigated the link between sleep dysfunction and psychotic experiences in groups identified as at ultra high risk of psychosis. Ruhrmann et al., 2010) utilised the sleep disturbance item from the Structured Interview for Prodromal Symptoms (SIPS; Miller et al., 1999) as one of six predictors in a model predicting transition to psychosis from a high risk state. This resulting model predicted transition to psychosis over 18 months with a sensitivity of only 41.7% but a high specificity of 97.9%. Lunsford-Avery et al. (2015) found that several actigraphically measured sleep variables (including decreased sleep efficiency and waking after sleep onset) were predictive of positive psychotic symptoms at baseline and 12 month follow up. An earlier cross-sectional study from the same authors on a similar sample (recording only self-reported sleep disruption) found an association with negative symptoms but not positive symptoms (Lunsford-Avery et al., 2013).

Retrospective reports similarly indicate that sleep dysfunction is notable before transition to first episode psychosis. Tan and Ang (2001) surveyed the most commonly reported prodromal symptoms across a group of first episode non-psychotic (major depression or anxiety disorders) and psychotic disorder inpatients at a military hospital. Sleep disruption was one of the most common symptoms reported in the prodrome of psychotic disorders (77%), although this is non-specific as sleep disruption was even more common (97%) in the non-psychotic clinical group.

Sleep disruption is also known to be a common symptom prior to relapse, accounting for its typical inclusion in scales developed to predict relapse in psychotic populations. The 34-item Early Signs Scale (ESS), which includes an item on recent sleep disruption, has demonstrated reasonable sensitivity (81%) and selectivity (79%) in identifying individuals who go on to relapse (Birchwood et al., 1989, Jørgensen, 1998a). The Warning Signals Scale, an shortened version of the ESS which retains self-reported sleep disruption as one of 8 items, also demonstrated acceptable specificity (68%) and selectivity (77%) in detecting individuals prior to relapse in delusions in a large sample of 131 patients (Jørgensen, 1998b). These findings suggest that the presence of sleep dysfunction is predictive of relapse into psychosis.

Sleep has also been linked to the broader well-being of individuals with psychosis. Several studies have indicated an association between the severity of positive psychotic symptoms and poorer sleep quality and quality of life (Afonso et al., 2011a, Afonso et al., 2014), including in a large sample (n = 505) of patients with schizophrenia (Xiang et al., 2009), although one smaller study (n = 145) found no direct link between self-reported sleep quality and positive symptoms (Ritsner, Kurs, Ponizovsky, & Hadjez, 2004). Reduced sleep duration (as indexed from clinical records during an inpatient stay) was found to be strongly associated with increased positive symptom severity within a group of patients with childhood-onset schizophrenia (Mattai et al., 2006).

From these studies it seems likely that sleep dysfunction among individuals with psychosis is associated with poorer clinical outcomes. The role of sleep disturbance in predicting transition to first episode psychosis or relapse is especially intriguing. However, the majority of studies on this topic to date do not attempt to measure the influence of sleep on individual psychotic experiences and the measure of sleep dysfunction is often a single self-report item, limiting further interpretation of this association.

#### 3.2.4 Sleep disorders and their relationship to psychotic experiences in clinical groups

Only five studies identified by the review directly investigated whether sleep disorders are associated with positive symptoms or individual psychotic experiences (Table 7a).

Freeman et al. (2009) studied the relationship between insomnia and paranoia in a sample of 30 patients with persecutory delusions and a community sample of 300 individuals. High rates of insomnia were seen in the clinical group: 54% of the individuals with persecutory delusions had moderate or severe insomnia, versus 7.4% of the community sample. Within each group dimensional measures of paranoia and insomnia severity were positively associated, with the relationship partly mediated by depression and anxiety. A larger study on outpatients with schizophrenia in Hong Kong and Beijing (n = 505) found that patients reporting one or more insomnia symptoms exhibited more severe positive symptoms than patients who slept well (Xiang et al., 2009). Severity of depressive symptoms was a significant contributor to poor sleep in this sample. In contrast with the above findings, Palmese et al. (2011) found no correlation between insomnia symptoms and positive symptoms — although insomnia prevalence was again found to be elevated in this group, with only 21% of participants reporting no significant sleep difficulties.

A high proportion of patients with psychotic disorders have also been found to experience weekly distressing nightmares: 55% (in comparison to a 0.9–6.8% estimated prevalence in the general population) (Sheaves, Onwumere, et al., 2015). The authors found that the level of distress associated with the nightmares correlated with the severity of delusions, with this association remaining significant after controlling for nightmare frequency. Another study reported no association between nightmare frequency and positive symptoms, although patients and individuals at high risk for psychosis did show elevated nightmare frequency compared to controls (Michels et al., 2014).

The above studies are only cross-sectional, and therefore can provide no indication about the direction of causal effect. However, four recently-published trials have begun to address the question of whether improving sleep in patients with schizophrenia would also reduce psychotic experiences, though all are substantially underpowered to address the connection adequately (Table 7b).

A double-blinded randomised controlled trial of a pharmacological treatment for insomnia (eszopiclone) in patients with schizophrenia found that positive psychotic symptoms showed greater reduction of insomnia symptoms in the treatment group (CI — 3.1; 0.4) versus the group receiving placebo (Tek et al., 2014). The confidence interval indicates the potential impact of the sleep intervention. While this difference was not statistically significant, such pilot trials are underpowered to produce effects significant at conventional levels (Lee, Whitehead, Jacques, & Julious, 2014). Furthermore only 39 of the intended sample of 80 were recruited, which will have further reduced the study power.

An open-label non-randomised trial of a pharmacological intervention (sodium oxybate) for insomnia on a small sample (n = 8) of inpatients with schizophrenia (Kantrowitz et al., 2010) found large effect size improvements in several PSG sleep variables (including an increase in SWS time and REM onset latency), an improvement in self-rated sleep quality, and a reduction in total Positive and Negative Symptom Scale score (PANSS; Kay, Fiszbein, & Opfer, 1987). However, there was no reported significant specific effect on the positive symptoms subscale, and the reported outcomes do not provide mean changes or confidence intervals.

A case series study of cognitive behavioural therapy for insomnia (CBTi) trial reported a clear improvement in psychotic experiences and sleep. 15 outpatients with persecutory delusions received 4 sessions of CBTi in an un-blinded uncontrolled trial (Myers et al., 2011). In this case, large effect size improvements in insomnia symptoms and persecutory delusions were observed with reductions also observed in anxiety, depression, and anomalies of experience. A subsequent single-blind randomised controlled trial testing the efficacy of CBT for sleep problems in 50 patients with persistent delusions and hallucinations has been completed (Freeman & Waite et al., 2015). CBT was highly efficacious in improving insomnia in this population (Cohen's d effect size = 1.9), but was inconclusive about the impact of this treatment on psychotic experiences because of the sample size.

#### 3.2.5 Sleep and treatment for psychosis

A number of studies indicate that improvement in symptoms following antipsychotic treatment is associated with improvement in sleep variables (Taylor et al., 1991a, Yamashita et al., 2004, Yamashita et al., 2005). Sleep dysfunction may also predict poorer clinical outcome following medication withdrawal (Chemerinski et al., 2002, Neylan et al., 1992), although in one smaller study sleep variables displayed no relationship with clinical symptoms (Nofzinger, van Kammen, Gilbertson, Gurklis, & Peters, 1993).

On balance such studies do appear to support an association between changes in positive psychotic symptoms and sleep. However, in this context it is difficult to associate any changes reported in sleep variables directly to changes in symptoms due to the likely direct of antipsychotic medication on sleep via their influence on multiple neurotransmitter systems (Cohrs, 2008). This effect particularly complicates interpretation of studies on the impact of withdrawing or changing antipsychotic medications such as those described here.

A recent randomised controlled trial indicated that improvement in positive psychotic symptoms following electroconvulsive therapy (ECT) is associated with an improvement in sleep variables (Zhang et al., 2012). The magnitude of reduction in positive symptoms following ECT significantly correlated with increases in sleep efficiency, REM onset latency, and REM density. Furthermore, sleep efficiency and positive psychotic symptoms improved further in the group which received ECT in addition to treatment as usual (antipsychotic medication) compared to a control group receiving only treatment as usual. The method of action of ECT on psychosis or sleep remains unclear (N. Kato, 2009) although it has been proposed that the mechanism of action of ECT is to mimic the EEG characteristics of slow wave/delta sleep (Charlton, 1999).

## 4 Discussion

There are surprisingly few studies that have assessed both individual psychotic experiences and sleep dysfunction. Currently the best quality evidence is in non-clinical populations, primarily utilising subjective reports of sleep. Here there is convincing evidence that psychotic experiences and sleep dysfunction co-occur (especially with respect to insomnia and paranoia). Yet this does not elucidate the direction of causal effects. There are fewer studies testing longitudinal data, but those that exist do indicate that sleep dysfunction (particularly symptoms of insomnia and nightmares) predicts later psychotic experiences. The opposite direction of causation (i.e. whether psychotic experiences predict later sleep dysfunction) remains to be tested. Evidence from manipulation studies, the strongest research design, is so far limited to sleep deprivation protocols using small sample sizes. Regardless, these studies do indicate that reducing sleep elicits psychotic-like experiences, as consistent with a causal role for sleep dysfunction in psychosis.

The clear next step for manipulation studies in the general population would be to test whether treatment of insomnia affects the occurrence of psychotic experiences. Such a study is already underway (Freeman & Sheaves et al., 2015). Another focus for future research in non-clinical populations would be to determine the mechanisms underlying the association between sleep and psychotic experiences. Anxiety and depression are consistently found to act as partial mediators, but these are typically the only potential mediating factors tested. Furthermore, assessment of sleep dysfunction should not always be restricted to insomnia given the broader association with other sleep disorders such as parasomnias reported here.

The studies in clinical populations are so far less conclusive. Issues include the heterogeneity of the populations studied (compounded by small sample sizes), and limited psychotic experiences assessment, although objective sleep assessments such as PSG and actigraphy have been widely applied — in contrast to the non-clinical studies. It seems likely that sleep dysfunction and psychotic experiences do co-occur, and that sleep might be a predictor of clinical change. The field requires much more attention. For instance, assessing a wide variety of sleep processes in relation to individual psychotic experiences at different stages in the clinical course of psychotic disorders. Substantial tests of a potential reciprocal relationship between sleep dysfunction and psychotic experiences are required. Further prospective studies are needed on the causal role that sleep problems may play in those at high risk of psychosis, particularly in relation to transition to first episode psychosis. The typical sleep changes in adolescence, in particular exhibiting a preference for a later chronotype (Gau and Soong, 2003, Gradisar et al., 2011, Keyes et al., 2015) are especially interesting in this regard, as this is the age at which psychotic experiences often begin to manifest. More thorough assessments of sleep at this period of elevated risk would be an advantage for future studies — in particular, considering individual sleep disorders rather than single item measures of sleep disturbance. As one example, a recent student population survey found that insomnia, nightmare frequency, and nightmare distress increased risk for mental illness in a dose–response fashion (Sheaves et al., in press).

Importantly, definitive studies of interventions to improve sleep in patients with psychotic experiences are needed. Again, this would ideally include tests of treating different types of sleep problems. For instance, there is a substantial case report literature going back more than 30 years on treatment of sleep apnoea in individuals with psychosis improving positive psychotic symptoms as well as sleep (e.g. Berrettini, 1980, Boufidis et al., 2003 January 1, Chiner et al., 2001, Karanti and Landén, 2007, Lee et al., 1989), yet no systematic trials have been undertaken to date (Kalucy et al., 2013, Waters et al., 2013). The small number of such studies, and the lack of any addressing disorders besides insomnia, is striking given guidance that co-morbid sleep disorders should receive treatment in their own right, and the existing indications that improving sleep may have the benefit of lessening psychotic experiences.

### 4.1 Theoretical integration

It is likely that further development in the understanding of the connection between sleep and psychosis could be made by integrating the findings with the existing theoretical literature in each of the two areas.

Previous studies drawing upon a cognitive model of persecutory delusions (Freeman et al., 2002) have shown that negative affect (anxiety and depression) may be an important link between sleep and paranoia. Another shared factor between this model of psychosis and cognitive models of insomnia that remains to be tested is the thinking style of worry. Other important routes – such as via anomalous experiences and reasoning biases – also remain to be investigated. Cognitive models of hallucinations, while yet to be explicitly linked with sleep, have similarly emphasised negative affect as having a key role in the source, form, and content of hallucinatory experiences (Waters et al., 2012). Furthermore, it is possible that sleep dysfunction could contribute to cognitive processes associated with hallucinatory experiences such as changes in source monitoring and inhibitory control. It is also possible that disturbances in sleep could mediate the relationship between trauma, PTSD, and hallucinatory experience (Morrison, Frame, & Larkin, 2003).

Parasomnias have historically received less attention than insomnia, yet indications from this review are that these disorders may have an important relationship to psychotic experiences, particularly in the case of nightmares. Dreams and nightmares have a longstanding association with psychosis owing to perceived phenomenological similarity (Limosani et al., 2011, Mason and Wakerley, 2012). The notion that in both states internally generated images or ideas are experienced as having an external origin, and as being intrinsically believable, has been carried through to modern theorising (Feinberg, 2011, Hobson, 2004). Cognitive models of nightmares (e.g. Nielsen & Levin, 2007) provide a link to models of paranoia and hallucinations via a shared emphasis on affective disturbance (in particular anxiety), as well as biases towards negative appraisals, for example of the nightmare event. Nightmares are most associated with REM sleep activity, which has been found to have an earlier onset and perhaps make up a higher percentage of total sleep time in psychotic populations — with both these characteristics correlating with increased positive psychotic symptom severity (Sarkar et al., 2010). An intriguing further direction for this research would be to directly investigate whether these REM sleep characteristics are associated with nightmare reports and psychotic experiences in clinical and non-clinical groups.

A neurodevelopmental model of psychosis, where psychosis is seen as resulting from aberrant synaptic pruning during adolescence, has been linked to sleep dysfunction in psychosis (Keshavan et al., 1994, Lunsford-Avery and Mittal, 2013). This account provides a plausible physiological underpinning for sleep dysfunction in psychosis, as structural abnormalities in key regions for promoting and maintaining sleep (e.g. the thalamus) are present in patients with first episode and chronic schizophrenia (Adriano, Spoletini, Caltagirone, & Spalletta, 2010), and in individuals who are at risk of schizophrenia (Lunsford-Avery et al., 2013). In the latter case the thalamic abnormalities were found to predict sleep dysfunction. Further work based on this model would need to establish a mechanism by which neurodevelopmental abnormalities or lack of sleep specifically lead to the experiences of individual psychotic experiences such as delusions or hallucinations.

Another body of research linking sleep and psychosis focuses on the impact of dysfunctional circadian rhythms in patients with schizophrenia. Studies utilising melatonin profiling in patients with schizophrenia have illuminated a range of circadian misalignments, such as delayed or advanced sleep phase, irregular sleep patterns, and free-running rest and activity patterns (where the circadian phase does not align to 24 h and therefore sleep and wake phases occur at different times each day) (Bromundt et al., 2011, Wulff et al., 2012). The melatonin profile in individuals with schizophrenia has been described as ‘blunted’, with reduced circadian variation in melatonin secretion, and a reduced correlation between rise in melatonin and sleep efficiency (Afonso, Figueira, & Paiva, 2011). Two small RCTs have indicated that melatonin supplements improve sleep quality in patients with schizophrenia (Kumar et al., 2007, Shamir et al., 2000) although neither investigated the effect of this treatment with psychotic experiences. A particular area of interest is the interaction of melatonin with dopamine and other key neurotransmitter systems associated with psychosis and the action of antipsychotic medications (Anderson and Maes, 2012, Monti et al., 2013). A range of animal studies have shown that melatonin inhibits dopamine release, increases dopamine turnover, and alters dopamine receptor activation (Monti et al., 2013), therefore one influence of a disturbed melatonin secretion profile could be the dopamine dysregulation thought to represent a ‘final common pathway’ to psychosis (Howes & Kapur, 2009). Again, further development based upon this model would need to specify mechanisms linked to individual psychotic experiences.

The salience model of psychosis (Kapur, 2003, Kapur et al., 2005) has been linked to sleep dysfunction via the proposed role of sleep in synaptic homeostasis (Tononi & Cirelli, 2014). According to the synaptic homeostasis hypothesis, the need for sleep (or sleep pressure) arises physiologically as a result of synaptic potentiation in wakefulness (new connections are constantly made in response to the environment while awake, but there is no corresponding downscaling or removing of associations). Slow wave sleep, in this theory, mediates a downscaling of synaptic strength, allowing informational efficiency to be restored for the next day. This would imply that, if sleep were disturbed as it is in psychosis, downscaling would be disrupted, and therefore the ability of the neural network to correctly detect novel associations may be disrupted. This would allow abnormal associations to arise and persist without contextual corrections, and may result in type 1-style errors in assessing the informational value (or salience) of these associations. Reports of individuals with psychosis being more likely to perceive meaning in ambiguous stimuli would support this account (Catalan et al., 2014, Galdos et al., 2011). On the other hand, the synaptic homeostasis hypothesis is primarily proposed as a theory of the function slow wave sleep, whereas the primary sleep architecture changes directly related to psychosis in the research literature to date involve REM sleep. Regardless, this theory does provide testable hypotheses for future research — for example, does sleep deprivation in non-clinical individuals cause similar changes in information processing? However the model does not differentiate how this process could contribute to the emergence of individual psychotic experiences, which have been shown to have independence from each other (e.g. Ronald, 2015).

A recent theory of positive psychotic symptoms provides a computational account based on Bayesian inference (Adams et al., 2013, Davies and Egan, 2013). Bayesian conceptualisations of psychosis describe delusions and hallucinations as both arising from faulty inference. A belief can be held with great precision or confidence — this corresponds to a narrowed variance in the probability distribution. In Bayesian inference, prior beliefs (or expectations) are transformed to posterior beliefs by combination with the current sensory evidence, its perceived accuracy, and the predicted likelihood of that sensory data occurring given the prior belief. If a piece of evidence is thought to be accurate, but unlikely given previous beliefs, these older beliefs will be altered. Such a system can be thought of as having confidence in the accuracy of an existing belief.

One model based on this theory proposes that psychosis results from a reduction of this confidence in relation to current sensory evidence (Adams et al., 2013). An impact of this would be that beliefs or perceptions are more malleable in the face of sensory evidence, i.e. there is a reduced influence of previous beliefs, due to their estimated low precision. One example of evidence for this model is that individuals with schizophrenia have been found to be less susceptible to visual illusions (Notredame, Pins, Deneve, & Jardri, 2014). As visual illusions work by capitalising on pre-existing beliefs about the environment, resistance to visual illusions in individuals with psychosis could be an expression of reduced influence of such beliefs. Some studies have reported that this reduced susceptibility to illusions correlates with the severity of positive psychotic symptoms (e.g. Silverstein et al., 2013) — and furthermore, sleep deprived non-clinical volunteers have been found to exhibit a similar resistance to visual illusions (Schmeider et al., 1996, Sternemann et al., 1997). Further development of this model would need to provide an account of how sleep dysfunction could contribute to this change in inferential processing — for example, whether sleep dysfunction could contribute to the dopaminergic modulations that have been suggested as instantiating this computational change in psychosis (Adams et al., 2013, Castelnovo et al., 2015). However, these models again have the issue of the absence of differentiation between different psychotic experiences.

In conclusion, we are beginning to see an acknowledgement of the potential role of sleep in the occurrence of psychotic experiences. It is now clear from the research that these two phenomena are connected. The direction of effect is yet to be shown and the mechanisms by which sleep and psychotic experiences are linked remain to be established, although there are many plausible different routes for further investigation. Even if future research indicates no causal connections between sleep and psychosis, we would argue that the high prevalence of sleep problems in patients with psychosis should still become a target in clinical services, since it is an important problem in its own right, for which effective treatments exist (Freeman & Waite et al., 2015). There is currently a striking absence of systematic research into the treatment of sleep problems in non-affective psychosis.

## Role of funding source

SR is a recipient of a Medical Research Council (MRC) Doctoral Studentship and a Balliol College Dervorguilla Scholarship (University of Oxford). DF is supported by a MRC Senior Clinical Fellowship (G0902308). DF and BS receive research support from a Wellcome Trust strategic award (098461/Z/12/Z) to the Oxford Sleep and Circadian Neurosciences Institute (SCNi).

## Role of contributors

SR conducted the systematic literature review and wrote a first draft under the supervision of DF and BS. DF and BS edited the text and contributed to the writing.

## Conflict of interest

None.

## Figures and Tables

**Table 1a:** Objective sleep measurement methods.

Sleep or circadian variable	Definition
Polysomnography (PSG)	Recording of electrical activity during sleep from multiple sources – typically including electroencephalography, electrooculography, electromyography, and electrocardiogram respectively monitoring electrical activity in the brain, eyes, muscles and heart. May also include respiratory indicators or other measurements.
Actigraphic monitoring	Continuous activity monitoring, usually carried out over several days via a device worn on the wrist, allows objective measurement of sleep-wake patterns (time to sleep, time to wake, sleep onset, night wakings etc.)
Melatonin concentration	Melatonin production rises during evening and peaks during the night. Irregular peaks or reduced amplitude in melatonin cycles are associated with circadian rhythm sleep-wake disorders (Table 2). Regular collection of saliva or urine allows measurement of concentrations of melatonin throughout the day, which can be combined with actigraphic monitoring to assess circadian functioning

**Table 1b:** Commonly assessed sleep variables.

Sleep or circadian variable	Definition
Sleep latency	Time from lights out until first occurrence of sleep
Sleep Period Time (SPT)	Time from sleep onset until final awakening, including intermittent waking periods
Total Sleep Time (TST)	Total time spent in any REM or NREM sleep stage
Sleep efficiency	Percentage of time in bed (TIB) spent asleep
REM onset latency	Time between sleep onset and the first occurrence of REM
REM density	Measure of frequency of rapid eye movements during REM sleep
Slow wave sleep	Amount of non-REM sleep stages 3 and 4 (also known as delta sleep)

**Table 2:** Sleep disorders and their symptoms.

Sleep disorder/symptom	Definition/key symptoms	Population prevalence (from DSM-V APA, 2013)
Insomnia	Difficulty initiating or maintaining sleep or nonrestorative sleep causing significant functional impairment.	6–10%
Sleep apnoea	A breathing related sleep disorder where breathing is obstructed during sleep, blocking off airflow and disturbing sleep. The primary symptoms are loud snoring and daytime sleepiness.	2–4%
Hypersomnia	Characterised by symptoms of excessive daytime sleepiness, and/or extended nocturnal sleep period.	1%
Parasomnias	A group of sleep disorders linked by abnormal behaviours, emotions, and dreams during sleep that lead to intermittent awakenings and difficulty resuming sleep.	Varies across disorder
Nightmare disorder	A disorder within the parasomnia group, characterised by frequent nightmares causing clinically significant distress or impairment in functioning — one or more episodes a week is classed as moderate severity.	6%
Circadian rhythm sleep–wake disorders	A group of sleep disorders resulting primarily from alteration of the circadian system or to misalignment between the endogenous circadian rhythm and the sleep-wake schedule required by an individual's environment. These disorders are split into types by the alteration seen for example — delayed sleep phase, advanced sleep phase and non-24 h sleep-wake type.	Varies across sub-type

**Table 3:** Studies addressing the link between sleep and psychotic experiences in non-clinical populations.

Citation	Design	N	Sample characteristics	Psychosis measure(s)	Sleep dysfunction measure(s)	Comment on findings	Linked?
(Fisher et al. (2014)	Longitudinal	6796	Children (aged 12) from population cohort (ALSPAC)	*Psychotic experiences* PLIKSi	*Sleep disorder symptoms* Postal questionnaires (completed by mothers) at age 2.5, 3.5, 4.75, 6.75, and 9 years. Sleep problems at 12 years assessed in semi-structured diagnostic-based interview with child.	Children with more frequent nightmares between 2.5 and 9 years more likely to report psychotic experiences at age 12. Children reporting any parasomnia at age 12 also found to have higher rates of psychotic experiences.	Y
Freeman et al. (2009)	Cross-sectional	300	Convenience sample from community	*Paranoia* G-PTS	*Insomnia symptoms* ISI, Sleep-50	Insomnia associated with paranoid thinking, association partly accounted for by anxiety and depression.	Y
Freeman, Brugha, et al. (2010)	Cross-sectional	8580	National epidemiological survey (British National Survey of Psychiatric Morbidity)	*Paranoia* PSQ, SCID-II	*Insomnia symptoms* CIS-R	Insomnia associated with an increase in paranoid thinking, association partly accounted for by affective symptoms or cannabis use.	Y
Freeman et al. (2011)	Cross-sectional	7281	National epidemiological survey (USA — Adult Psychiatric Morbidity Survey)	*Paranoia* Paranoia items from survey (3)	*Insomnia symptoms* CIS-R	Paranoia associated with insomnia, odds ratios of insomnia diagnosis increases with level of paranoia.	Y
Freeman et al. (2012)	Longitudinal	2382	National epidemiological survey (British Psychiatric Morbidity Survey 2000)	*Paranoia* PSQ (3 items) SCID-II (at baseline and 18 month follow up)	*Insomnia symptoms* CIS-R (at baseline and 18 month follow up)	Insomnia symptoms predicted new paranoid thinking or persistence of existing paranoid thinking at 18 month follow-up.	Y
Freeman et al. (2013)	Longitudinal	106	Individuals attending hospital after an assault	*Paranoia* G-PTS SSPS PANSS PSYRATS 4 paranoia and threat related visual analogue scales (all at baseline and 6 month follow up)	*Insomnia symptoms* ISI (at baseline and 6 month follow up)	Insomnia at baseline was found to predict paranoia (G-PTS) and post-traumatic stress disorder symptoms at 6 month follow up. Current insomnia was also a significant predictor of paranoia scores at 6 months.	Y
Hurdiel et al. (2014)	Manipulation (sleep deprivation)	17	Volunteers completing ultramarathon event	*Psychotic experiences* Hallucinations — open ended question on completion	*Objective sleep* Wrist watch actigraphy throughout event *Sleep deprivation* Average 46 h 38 min	4 out of 17 participants reported experiencing hallucinations during the exercise event.	Y
Jeppesen et al. (2014)	Cross-sectional	1623	Adolescents (aged 11/12 years) from population cohort	*Psychotic experiences:* K-SADS-PL	*Subjective sleep* Self-report of sleep disturbance in structured interview	Likelihood of psychotic experiences increased with sleep problems and emotional or neurodevelopmental disorders. Increased prevalence of sleep disorders among children with psychotic experiences.	Y
Kahn-Greene et al. (2007)	Manipulation (sleep deprivation)	25	Non-clinical volunteers (recruited from military)	*Psychotic experiences* PAI (pre and post sleep deprivation)	*Sleep deprivation* 56 h	Sleep deprivation resulted in increase in anxiety, depression and paranoia, but not manic-related symptoms or schizophrenia symptom factors.	Y
Koyanagi and Stickley (2015)	Cross-sectional	267,692	WHO population based survey (56 countries)	*Psychotic experiences* Single items from CIDE for delusional mood, delusions of persecution/reference, delusions of control, hallucinations	*Insomnia symptoms* 1 item for presence of insomnia symptoms (5 point scale of ‘none’ to ‘extreme’)	Sleep problems associated in dose–response fashion with psychotic symptoms in almost all countries with significant ORs from 2.26–2.84 (1.54–1.68 after controlling for anxiety and depression).	Y
Lee et al. (2012)	Cross-sectional	7172	23 high school cohorts of students (aged 12–17)	*Psychotic- experiences* ESI *Psychosis risk:* Y-PARQ	*Insomnia* Self report questionnaire modelled on ICD-10 diagnostic criteria for insomnia *Excessive Daytime Sleepiness* ESS *Cataplexy* Single item, muscle weakness *Snoring* Single item, frequency of snoring	Insomnia and excessive daytime sleepiness predicted psychotic like experiences (and higher risk scores for psychosis) in adolescent group, independent of depressive symptoms.	Y
Levin and Fireman (2002)	Cross-sectional	116	Undergraduate psychology students selected for high nightmare prevalence (at least 3–10 a year) from screening survey	*Psychoticism/paranoia* SCL-90-R	*Nightmares* Dream log	Nightmare distress (not frequency) associated with paranoia, anxiety, depression. Nightmare frequency associated with psychoticism scale score.	Y
Oshima et al. (2010)	Cross-sectional	a) 279 b) 62	High school cohort of students (aged 12–17) a) singletons b) twin students (31 pairs)	*Psychotic- experiences* Custom self-report questionnaire (4 items)	*Subjective sleep* GHQ-12	Poor sleep associated with psychotic like experiences.	Y
Petrovsky et al. (2014)	Manipulation (sleep deprivation)	24	Student volunteers	*Psychotic- experiences* PSI (pre and post sleep deprivation)	*Sleep deprivation* Overnight sleep deprivation *Sleepiness* SSS at 9 pm and from 7 to 10 am following morning (6 time points in total)	Sleep deprivation induced perceptual distortions, cognitive disorganisation and anhedonia, but not mania, paranoia or delusional thinking.	Y
(Sheaves, Bebbington, et al., submitted)	Longitudinal	a) 8580 b) 7403 c) 2046	National epidemiological surveys: a) British Psychiatric Morbidity Survey 2000 b) British Psychiatric Morbidity Survey 2007 c) British Psychiatric Morbidity Survey 2000 18 month follow up	*Paranoia and hallucinations* PSQ (2 items) (at baseline and 18 month follow up)	*Insomnia symptoms* CIS-R (at baseline and 18 month follow up)	Insomnia predicted new hallucinatory experiences at 18 month follow up. Presence of insomnia raised likelihood of reporting hallucinations cross-sectionally. All remained significant when controlling for depression, anxiety and paranoia.	Y
Taylor et al. (2015)	Cross-sectional	a) 5076 b) 5059	a) Twin pairs (aged 16) from population cohort b) parents of twins	*Psychotic- experiences* SPEQ	*Subjective sleep* PSQI *Insomnia symptoms* ISI	Shared genetic and environmental mechanisms for psychotic experiences and sleep disturbances – association reduced but remained significant after controlling for negative affect.	Y
Thompson et al. (2015)	Longitudinal	4270	18 year old population cohort sample (ALSPAC)	*Psychotic- experiences* *PLIKSi* (at age 12 and 18)	*Sleep disorder symptoms* Postal questionnaires (completed by mothers) at age 2.5, 3.5, 4.75, 6.75, and 9 years. Sleep problems at 12 years assessed in semi-structured diagnostic-based interview with child.	Nightmares at age 12 a significant predictor of psychotic experiences at age 18, remaining after adjustment for mood and other confounders.	Y

CIDI = (Kessler & Ustün, 2004); CIS-R = Clinical Interview Schedule (revised) (Lewis, Pelosi, Araya, & Dunn, 1992); ESI = Eppendorf Schizophrenia Inventory (Mass, 2000); ESS = Epworth Sleepiness Scale (Johns, 1991); GHQ-12 = 12 item version of the General Health Questionnaire (Goldberg & Williams, 1988); G-PTS = Green et al. paranoid thoughts scale (Green et al., 2008); ISI = Insomnia Severity Index (Morin, 1993); K-SADS-PL- Schedule for schizophrenia and affective disorders for school-age children, present and lifetime edition (Kaufman et al., 1997); PAI = Personality Assessment Inventory (Morey, 1991); PANSS = Positive and Negative Symptom Scale (Kay et al., 1987); PLIKSi = Psychosis-Like Symptom Interview (Horwood et al., 2008); PSI = Psychotomimetic States Inventory (Mason et al., 2008); PSQ = Psychosis Screening Questionnaire (Bebbington & Nayani, 1995); PSQI = Pittsburgh Sleep Quality Index (Buysse, Reynolds, Monk, Berman, & Kupfer, 1989); PSYRATS = Psychotic symptom rating scales (Haddock, McCarron, Tarrier, & Faragher, 1999); SCID-II = Structured Clinical Interview for DSM-IV disorder (First, Gibbon, Spitzer, Williams, & Benjamin, 1997); SCL-90-R = Symptom Checklist 90 (Revised) (Derogatis, 1994); SLEEP-50 = (Spoormaker, Verbeek, van den Bout, & Klip, 2005); SPEQ = Specific Psychotic Experiences Questionnaire (Ronald et al., 2014); SSPS = State Social Paranoia Scale (Freeman et al., 2007); SSS = Stanford Sleepiness Scale (Hoddes, Zarcone, Smythe, Phillips, & Dement, 1973); Y-PARQ = Youth Psychosis at Risk Questionnaire (Ord, Myles-Worsley, Blailes, & Ngiralmau, 2004).

**Table 4:** Studies addressing the link between positive symptoms and sleep measured with polysomnography in clinical groups.

Citation	N	Sample characteristics	Psychosis measure(s)	# PSG nights	Comment on findings	Linked?
Benson and Zarcone (1993)	a) 21 b) 24 c) 13	a) Patients with schizophrenia (at least 2 weeks medication free) b) Patients with major depressive disorder c) Non-clinical controls	*Positive symptoms* BPRS	2	No significant differences in REM eye movements across groups, one positive correlation for a hallucinatory behaviour item and REM density in the schizophrenia group.	Y (partly)
Ferrarelli et al. (2010)	a) 49 b) 20 c) 44	a) Patients with schizophrenia b) Patients with other diagnoses receiving antipsychotic medication c) Non-clinical controls	*Positive symptoms* PANSS	1	Negative correlation between sleep spindle activity and number and positive symptoms.	Y
Ganguli (1987)	a) 8 b) 8 c) 16 d) 16 e) 10	a) Patients with delusional depression b) Depression c) Non-clinical controls	*Positive symptoms* BPRS	2	Positive correlation between REM latency and severity of psychosis, but trend failed to reach statistical significance.	Y (partly)
Kajimura et al. (1996)	a) 6 b) 6	a) Patients with schizophrenia (outpatients) b) Non-clinical controls	*Positive symptoms* BPRS	1	Negative correlation between slow wave sleep and negative symptom scale. No associations reported between PSG variables and positive symptoms.	N
Keshavan et al. (1995)	a) 24 b) 5 c) 4 d) 5	Patients with: a) Schizophrenia b) Schizoaffective bipolar c) Schizoaffective depressed d) Delusional disorder	*Positive symptoms* BPRS SAPS	2 or 3	Negative correlation between slow wave sleep and negative symptom scale. No association reported between SWS and positive symptoms.	N
Keshavan et al. (1998)	a) 30 b) 30	c) Unmedicated patients with schizophrenia (medication naïve or medication free for average of 40 weeks) d) Non-clinical age and sex matched controls	*Positive symptoms* BPRS	2 or 3	Patients with schizophrenia had reduced slow wave sleep. No correlations between positive symptoms and slow wave sleep.	N
Lauer et al. (1997)	a) 22 b) 20	a) Drug naïve, first episode or acute exacerbation patients with schizophrenia b) Non-clinical age and sex matched controls	*Positive symptoms* BPRS	1	Positive symptoms scores correlate negatively with REM onset latency, but reported to be driven by conceptual disorganisation factor rather than all positive symptom factors.	Y
Manoach et al. (2014)	a) 26 b) 25 c) 19 d) 23	a) Patients with schizophrenia (inpatient and outpatient, n = 11 early course) b) Non-clinical age and sex matched controls (to patient group) c) First degree relatives of patients with schizophrenia d) Non-clinical age, sex and education matched controls (to relatives group)	*Positive symptoms* SAPS	1	Positive correlation increased amplitude of spindles during stage 2 sleep and positive symptoms (only found in patients with schizophrenia).	Y
Poulin et al. (2003)	a) 11 b) 11	a) Patients with first episode psychosis (medication naïve) b) Non-clinical controls	*Positive symptoms* BPRS	1 or 2	Positive symptom scores correlated negatively with REM onset latency. Other sleep measures correlate with total symptom severity.	Y
Rotenberg et al. (1997)	20	Patients with schizophrenia (medicated)	*Positive symptoms* PANSS	3	Positive symptoms correlated with lowered REM density.	Y
Sarkar et al. (2010)	a) 20 b) 14 c) 20	a) Patients with schizophrenia b) First degree relatives of patients c) Non-clinical age and sex matched controls	*Positive symptoms* BPRS PANSS	1	Positive symptoms correlated positively with percentage of time in REM sleep, and negatively correlated with REM sleep onset latency.	Y
Tandon et al. (1989)	10	Patients with schizophrenia (inpatients)	*Positive symptoms* BPRS	1	No difference in BPRS score between patients with and without REM abnormalities.	N
Tandon et al. (1992)	a) 20 b) 20 c) 15	a) Drug-naïve schizophrenia patients b) Previously medicated but drug free schizophrenia patients c) Non-clinical controls	*Positive symptoms* BPRS	1	Positive symptoms scores correlate negatively with REM onset latency, but effect only found in previously treated group, not in the drug naïve group.	Y (partly)
Taylor, Tandon, Shipley, Eiser, et al. (1991)	36	Patients with schizophrenia (inpatients)	*Positive symptoms* BPRS	1	No difference in BPRS positive scale score in patients with REM at sleep onset (n = 6) versus patients without REM at sleep onset (n = 30).	N
Tekell et al. (2005)	a) 17 b) 15 c) 17	a) Patients with schizophrenia b) Patients with major depression c) Non-clinical controls	*Positive symptoms* BPRS SAPS	1	Positive symptom scores explained significant amount of variance in high frequency EEG activity during REM sleep.	Y
Tesler et al. (2015)	a) 9 b) 15	a) Adolescents (aged 14–18) meeting criteria for early onset schizophrenia spectrum disorder b) Non-clinical age and sex matched controls	*Positive symptoms* PANSS	1	Reduced sleep spindle density in clinical group correlated with positive symptom severity.	Y
Wamsley et al. (2012)	a) 21 b) b) 17	a) Chronically medicated patients with schizophrenia b) Non-clinical age and sex matched controls	*Positive symptoms* PANSS	1	Reduced amplitude and power of individual spindles correlated with greater severity of positive symptoms.	Y
Yang and Winkelman, (2006)	a) 15 b) 15	a) Inpatients with schizophrenia (at least 2 weeks drug free) b) Non-clinical controls	*Positive symptoms* BPRS	1	Positive symptoms correlated with lowered REM density.	Y

BPRS = Brief Psychiatric Rating Scale (Overall & Gorham, 1962); SAPS = Scale for Assessment of Positive Symptoms (Andreasen, 1984); PANSS = Positive and Negative Symptom Scale (Kay et al., 1987).

**Table 5:** Studies addressing the link between positive psychotic symptoms and sleep measured with actigraphy in clinical groups.

Citation	Design	N	Sample characteristics	Psychosis measure(s)	Sleep dysfunction measure(s)	Comment on findings	Linked?
Afonso, Brissos, et al. (2011)	Cross sectional	23	Patients with schizophrenia (outpatients)	*Positive symptoms* PANSS (used to divide into predominant positive (n = 11) and predominant negative (n = 12) groups)	*Subjective sleep* PSQI *Circadian sleep measures* Wrist actigraphy (7 days continuous)	Patients with more positive symptoms report reduced sleep quality and quality of life in comparison to patients with predominantly negative symptoms.	Y
Afonso et al. (2014)	Cross sectional	a) 34 b) 24	a) Patients with schizophrenia (outpatients) b) Age and gender matched non-clinical controls	*Positive symptoms* PANSS	*Subjective sleep* PSQI *Circadian sleep measures* Wrist actigraphy	Increased positive symptoms associated with reduced sleep quality and more disturbed sleep-wake patterns in patient group.	Y
Bromundt et al. (2011)	Cross-sectional	14	Patients with schizophrenia (outpatients)	*Positive symptoms* PANSS	*Subjective sleep* PSQI *Circadian sleep measures* Wrist actigraphy (21 days) Melatonin profiling (48 h window collected weekly)	No relationship found between positive symptoms and cognitive performance or sleep-wake measures.	N
Lunsford-Avery et al. (2015)	Longitudinal	a) 36 b) 31	a) Ultra high risk youth b) Non-clinical controls	*Psychotic symptoms* SIPS SCID (both at baseline and 12 month follow up)	*Circadian sleep measures* Wrist actigraphy *Subjective sleep* PSQI	Sleep disturbance significantly associated with increased positive symptoms at baseline in high risk group, and predict clinical symptoms at 12 months when controlling for age, depression and baseline psychotic symptoms.	Y
Martin et al. (2001)	Cross-sectional	28	Older patients with schizophrenia (mean age = 58 yrs)	*Positive symptoms* BPRS SAPS	*Objective sleep* Wrist actigraphy (activity and light exposure – 3 days)	No clinical or demographic variables were related to sleep wake characteristics.	N
Waters et al. (2011)	Longitudinal	a) 6 b) 7	a) Patients with schizophrenia (outpatients) b) Age and gender matched non-clinical controls	*Positive symptoms* BPRS *Psychotic experiences* 12 item questionnaire including 8 items on psychotic experiences, 4 on mood (daily)	*Subjective sleep* PSQI *Circadian measures* Wrist actigraphy (up to 28 days)	Sleep variations in the clinical group predicted daily changes in positive symptoms and negative mood.	Y
Wichniak et al. (2011)	Cross-sectional	73	Patients with schizophrenia spectrum disorder (treated with olanzapine or risperidone)	*Positive symptoms* PANSS	*Objective sleep* Wrist actigraphy (7 days)	Positive symptom severity correlated with reduced activity.	Y
Wulff et al. (2012)	Cross sectional	a) 20 b) 21	a) Patients with schizophrenia (outpatients) b) Age, gender and employment matched non-clinical controls	*Positive symptoms* Clinician scored presence or absence of positive symptoms	*Subjective sleep* PSQI *Circadian measures* Wrist actigraphy Melatonin profiling (48 h window collected weekly)	Presence or absence of positive symptoms did not predict differences in circadian sleep/wake disruptions.	N

BPRS = Brief Psychiatric Rating Scale (Overall & Gorham, 1962); PANSS = Positive and Negative Symptom Scale (Kay et al., 1987); PSQI = Pittsburgh Sleep Quality Index (Buysse et al., 1989); SCID = Structured Clinical Interview for DSM-IV Disorder (First et al., 1997); SIPS = Structured Interview for Prodromal Symptoms (Miller et al., 1999).

**Table 6:** Studies addressing the link between sleep and clinical course of psychosis.

Citation	Design	N	Sample characteristics	Psychosis measure(s)	Sleep dysfunction measure(s)	Comment on findings	Linked?
Afonso, Brissos, et al. (2011)	Cross sectional	23	Patients with schizophrenia (outpatients)	*Positive symptoms* PANSS (used to divide into predominant positive (n = 11) and predominant negative (n = 12) groups)	*Subjective sleep quality* PSQI Circadian sleep measures Wrist actigraphy (7 days continuous)	Patients with more positive symptoms report reduced sleep quality and quality of life in comparison to patients with predominantly negative symptoms.	Y
Afonso et al. (2014)	Cross sectional	a) 34 b) 24	a) Patients with schizophrenia (outpatients) b) Age and gender matched non-clinical controls	*Positive symptoms* PANSS	*Subjective sleep* PSQI *Circadian measures* Wrist actigraphy	Negative correlation found between positive symptoms and self-reported sleep quality in patient group.	Y
Birchwood et al. (1989)	Longitudinal	19	Patients with schizophrenia (outpatients)	*Positive symptoms* PAS (repeated monthly) Relapse (defined as readmission or incipient readmission)	*Subjective sleep* ESS (repeated every 2 weeks for 18 weeks)	Sleep disruption item formed part of self-report scale predicting relapse — sleep disruption reported prior to positive symptoms on PAS.	Y
Jørgensen (1998a)	Longitudinal	60	Patients with schizophrenia (outpatients)	*Positive symptoms* Psychotic scale of PANSS (repeated every 2 weeks for 6 months or to relapse), relapse defined as increase in 2 in any item	*Subjective sleep* ESS (repeated every 2 weeks for 6 months or to relapse)	Increase of 10 points on ESS predicted relapse with sensitivity of 81% and selectivity of 79%.	Y
Jørgensen (1998b)	Longitudinal	131	Patients with schizophrenia (outpatients)	*Positive symptoms* Psychotic scale of PANSS (repeated every 2 weeks), delusion relapse defined as increase of 2 in delusions scale	*Subjective sleep* WSS (repeated every 2 weeks for 6 months or to relapse)	8-item Warning Signals Scale (condensed from ESS found to have sensitivity of 77% and specificity of 68% in detecting delusion formation.	Y
Kim, Lee, Kim, Jung, and Lee (2009)	Retrospective	20	Patients with first episode psychosis	*Psychotic symptoms* K-NOS (Korean version)	*Subjective sleep* K-NOS (Korean version)	Sleep changes reported in prodrome of 16–43% of patients.	Y
Lunsford-Avery et al. (2013)	Cross sectional	a) 33 b) 33	a) Adolescents (aged 12–21 years) b) Satisfying UHR criteria c) Age matched non-clinical controls	*Prodromal positive symptoms* SIPS SCID	*Subjective sleep* PSQI	Sleep dysfunction associated with negative symptom severity but not positive symptoms.	N
Lunsford-Avery et al. (2015)	Longitudinal	a) 36 b) 31	a) Ultra high risk youth b) Non-clinical controls	*Psychotic symptoms* SIPS SCID (both at baseline and 12 month follow up)	*Objective sleep* Wrist actigraphy *Subjective sleep* PSQI	Sleep disturbance associated with increased positive symptoms at baseline in high-risk group, and predict clinical symptoms at 12 months when controlling for age, depression and baseline psychotic symptoms.	Y
Martin et al. (2001)	Cross-sectional	28	Older patients with schizophrenia (mean age = 58 yrs)	*Positive symptoms* BPRS SAPS	*Objective sleep* Wrist actigraphy (activity and light exposure)	No clinical or demographic variables were related to sleep wake characteristics.	N
Mattai et al. (2006)	Manipulation (observed over medication wash out period of 5–7 days)	61	Patients with childhood onset schizophrenia (mean age = 10 yrs)	*Positive symptoms* BPRS SAPS	*Sleep duration* Nursing notes and safety records — used to divide sample into good sleepers (> 6 h sleep) and poor sleepers (< 6 h sleep)	Poor sleepers had significantly increased positive symptoms scores in comparison to good sleepers.	Y
Ritsner et al. (2004)	Cross sectional	145	Patients with schizophrenia (inpatient and outpatient)	*Positive symptoms* PANSS	*Subjective sleep* PSQI — used to split sample into good sleepers (< 5, n = 79) and bad sleepers (> = 5, n = 66))	No significant difference in positive or negative symptoms across the good sleeper and poor sleeper groups.	N
Ruhrmann et al. (2010)	Longitudinal	245	Youth satisfying ultra high risk criteria or basic symptom based criterion cognitive disturbances	*Prodromal positive symptoms* SIPS	*Subjective sleep* SIPS (sleep disturbance item);	Model predicting transition from high risk status includes sleep disturbance as one of 6 predictors, predicts 18 month outcomes with sensitivity of 41.7% and specificity of 97.9%.	Y
Tan and Ang (2001)	Retrospective	a) 30 b) 34	Patients in military hospital with: a) first episode psychosis b) first episode non-psychotic and non-organic psychiatric conditions	*Positive symptoms* Unstructured and semi-structured interviews of patients and informants	*Subjective sleep* Unstructured and semi-structured interviews of patients and informants	Sleep disruption seen in prodrome of 77% of psychotic patients, but 97% of non-psychotic.	Y
Xiang et al. (2009)	Cross sectional	505	Outpatients with schizophrenia	*Positive symptoms* BPRS	*Insomnia symptoms* 3 DSM-IV based items — classification as ‘poor sleeper’ following positive answer to 1 item or more	36% of sample classified as ‘poor sleepers’. Poor sleep significantly associated with positive symptom severity and reduced quality of life.	Y

BPRS = Brief Psychiatric Rating Scale (Overall & Gorham, 1962); ESS = Early Signs Scale (Birchwood et al., 1989); G-PTS = Green et al. Paranoid Thoughts Scale (Green et al., 2008); ISI = Insomnia Severity Index (Morin, 1993); K-NOS = Korean version of the Nottingham Interview Schedule (Kim et al., 2009); PANSS = Positive and Negative Symptom Scale (Kay et al., 1987); PAS = Psychiatric Assessment Scale (Krawiecka, Goldberg, & Vaughan, 1977); PSQI = Pittsburgh Sleep Quality Index (Buysse et al., 1989); SAPS = Scale for Assessment of Positive Symptoms (Andreasen, 1984); PSYRATS = Psychotic Symptom Rating Scales (Haddock et al., 1999); SCID = Structured Clinical Interview for DSM-IV Disorder (First et al., 1997); SIPS = Structured Interview for Prodromal Symptoms (Miller et al., 1999); SLEEP-50 = (Spoormaker et al., 2005); WSS = Warning Signals Scale (Jørgensen, 1998b).

**Table 7a:** Studies addressing the link between sleep disorders or sleep disorder symptoms and psychosis.

Citation	Design	N	Sample characteristics	Psychosis measure(s)	Sleep dysfunction measure(s)	Comment on findings	Linked?
Freeman et al. (2009)	Cross sectional	a) 300 b) 30	a) Non-clinical community sample b) Individuals with persecutory delusions and diagnosis of non-affective psychosis.	*Paranoia* G-PTS	*Insomnia symptoms* ISI Sleep-50	Insomnia associated with paranoid thinking, relationship largely accounted for by anxiety and depression	Y
Michels et al. (2014)	Cross-sectional	a) 17 b) 14 c) 17 d) 29	a) Inpatients with schizophrenia b) ARMS outpatients c) Non-clinical relatives of patients with schizophrenia d) Non-clinical controls	*Positive symptoms* PANSS	*Nightmares* Frequency scale (past 2 months)	Positive symptoms did not correlate with nightmare frequency, although nightmares were more common in ARMS outpatients and schizophrenic groups.	N
Palmese et al. (2011)	Cross-sectional	175	Patients with schizophrenia or schizoaffective disorder (outpatients)	*Positive symptoms* CGI	*Insomnia symptoms* ISI PSQI *Night eating* NEQ	No significant relationship between positive symptom score and insomnia severity. No relationship reported between night eating and positive symptom score	N
(Sheaves, Onwumere, et al., 2015)	Cross sectional	40	Patients with psychotic symptoms (inpatient and outpatient)	*Psychotic experiences* PSYRATS	*Subjective sleep* PSQI *Nightmares* Dream log	High proportion of sample reported weekly distressing nightmares, distress associated with severity of delusions.	Y
Xiang et al. (2009)	Cross sectional	505	Outpatients with schizophrenia	*Positive symptoms* BPRS	*Insomnia symptoms* 3 DSM-IV based questions —classified as ‘poor sleeper’ following positive screen for > = 1	36% of sample classified as ‘poor sleepers’. Poor sleep significantly associated with positive symptom severity and reduced quality of life.	Y

ARMS = At Risk Mental State (Rausch et al., 2013); BPRS = Brief Psychiatric Rating Scale (Overall & Gorham, 1962); CGI = Clinical Global Impression Scale (Haro et al., 2003); G-PTS = Green et al. Paranoid Thoughts Scale (Green et al., 2008); ISI = Insomnia Severity Index (Morin, 1993); NEQ = Night Eating Questionnaire (Allison et al., 2008); PSQI = Pittsburgh Sleep Quality Index (Buysse et al., 1989); PSYRATS = Psychotic Symptom Rating Scales (Haddock et al., 1999); SLEEP-50 = ((Spoormaker et al., 2005).

**Table 7b:** Studies addressing the link between positive psychotic symptoms and treatment for sleep disorders.

Citation	N	Sample characteristics	RCT	Treatment	Psychosis measure(s)	Sleep dysfunction measure(s)	Comment on findings
(Freeman & Waite et al., 2015)	50	Outpatients with persistent persecutory delusions/hallucinations and insomnia	Y	CBT for insomnia (CBTi — 8 sessions in max 12 weeks)	*Psychotic experiences* PSYRATS PANSS (pre-treatment, post treatment, 12 week follow up)	*Insomnia symptoms* ISI (pre-treatment, post treatment, 12 week follow up)	CBT for insomnia led to large effect size reductions in insomnia symptoms. Direction of changes to delusions and hallucinations unclear.
Kato et al. (1999)	7	Male outpatients with schizophrenia	N	Change to insomnia medication (benzodiazapene to 8 weeks of zopiclone	*Positive symptoms* BPRS (pre and post treatment)	*Objective sleep* Polysomnography (pre and post treatment)	Slow wave sleep improved following move to zopiclone treatment, negative symptoms significantly improved (positive symptoms improved but non-significant)
Kantrowitz et al. (2010)	8	Inpatients with schizophrenia and insomnia	N	Insomnia medication (sodium oxybate — 4 weeks)	*Positive symptoms* PANSS (pre and post-treatment)	*Subjective sleep* PSQI (baseline and post-treatment) ESS (baseline and post-treatment) *Objective sleep* PSG (2 nights at baseline and last nights of treatment)	Pharmaceutical treatment of insomnia improved sleep (large improvements in SWS time, reduced sleep latency, and increased REM latency) and positive symptoms
Myers et al. (2011)	15	Outpatients with persistent persecutory delusions and insomnia	N	CBTi — 4 sessions, over max 8 weeks	*Paranoia* G-PTS *Psychotic symptoms* PSYRATS *Anomalous perceptions* CAPS (All at pre-treatment, post-treatment, and one month follow up)	*Insomnia symptoms* ISI *Subjective sleep* PSQI (All at pre-treatment, post-treatment, and one month follow up)	CBTi led to improvements in insomnia symptoms and persecutory delusions, with reductions also observed in anxiety, depression, and anomalies of experience.
Tek et al. (2014)	39	Clinically stable outpatients with schizophrenia and insomnia	Y	Insomnia medication (eszopiclone — 8 weeks)	*Positive symptoms* PANSS (every 2 weeks)	*Insomnia symptoms* ISI (weekly) *Subjective sleep* Sleep diary (every day)	Psychotic symptoms showed greater reduction in the treatment group (CI — 3.1; 0.4) compared to the group receiving placebo.

CAPS = Cardiff Anomalous Perceptions Scale (Bell, Halligan, & Ellis, 2006); ESS = Epworth Sleepiness Scale (Johns, 1991); G-PTS = Green et al. Paranoid Thoughts Scale (Green et al., 2008); ISI = Insomnia Severity Index (Morin, 1993); PANSS = Positive and Negative Symptom Scale (Kay et al., 1987); PSQI = Pittsburgh Sleep Quality Index (Buysse et al., 1989); PSYRATS = Psychotic Symptom Rating Scales (Haddock et al., 1999).

**Table 8:** Studies addressing the link between treatment of positive psychotic symptoms and sleep.

Citation	N	Sample characteristics	Treatment manipulation	Psychosis measure(s)	Sleep dysfunction measure(s)	Comment on findings	Linked?
Chemerinski et al. (2002)	122	Patients with schizophrenia	Withdrawal of antipsychotic medication	*Positive symptoms* SAPS (weekly)	*Insomnia symptoms* HAM-D insomnia items (weekly)	Patients reporting insomnia prior to withdrawal had more severe psychotic symptoms during medication-free period	Y
Neylan et al. (1992)	18	Clinically stable patients with schizophrenia inpatients	Withdrawal of antipsychotic medication	*Positive symptoms* Bunney–Hamburg (daily)	*Objective sleep* PSG (3 nights pre and post withdrawal)	Psychosis scale score correlated with REM time, inversely correlated with REM onset latency. This did not predict status following withdrawal of antipsychotic Increased symptoms after withdrawal were associated with reduced sleep continuity and decrease in total REM and NREM time.	Y
Nofzinger et al. (1993)	10	Male patients with schizophrenia	Withdrawal of antipsychotic medication	*Positive symptoms* BPRS (at 2 weeks and 6 weeks post withdrawal)	*Objective sleep* PSG (3 nights at 2 weeks and 6 weeks post withdrawal)	Changes in EEG sleep during withdrawal period did not correlate with positive symptoms.	N
Taylor, Tandon, Shipley, and Eiser, (1991)	14	Inpatients with schizophrenia	Administered antipsychotic medication (medication free at baseline)	*Positive symptoms* BPRS (pre-treatment and after average of 24.4 days of treatment)	*Objective sleep* PSG (one night pre-treatment and two nights after average of 24.4 days of treatment)	Positive symptoms correlate with shortened REM latency prior to antipsychotic treatment, but no symptom measure and sleep variable correlations found after treatment.	Y
Yamashita et al. (2004)	92	Inpatients with schizophrenia — same sample as (Yamashita et al., 2005), average age 61.5	Observed over change from conventional to atypical antipsychotics	*Positive symptoms* PANSS (baseline and 8 weeks post change completion)	*Subjective sleep* PSQI (baseline and 8 weeks post change completion)	Improvement in negative symptoms significantly correlated with improvement in PSQI scores, trend correlation (p = 0.08) with positive symptoms following medication switch.	Y
Yamashita et al. (2005)	a) 35 b) 51	Inpatients with schizophrenia: a) Elderly (age ≥ 65) b) Middle-aged (age < 65)	Observed over change from conventional to atypical antipsychotics	*Positive symptoms* PANSS (baseline and 8 weeks post change completion)	*Subjective sleep* PSQI (baseline, and 8 weeks post completion of change)	Improvement in sleep satisfaction was significantly correlated with improvement in all subscales of the PANSS (including positive symptoms), but only in the middle aged group. Correlation limited to negative symptoms and self-rated sleep quality in elderly group.	Y
Zhang et al. (2012)	a) 40 b) 18	First episode psychosis patients	a) Electroconvulsive therapy and antipsychotic medication b) Antipsychotic medication only	*Positive symptoms* PANSS (baseline and then repeated weekly during hospital stay)	*Objective sleep* PSG (overnight, recorded at baseline and week 2)	Reduction in positive symptoms significantly correlated with improvements in sleep efficiency, REM latency, and REM density. Sleep efficiency and positive symptoms improved further from baseline in the ECT and antipsychotic group versus the antipsychotic only group.	Y

SAPS = Scale for Assessment of Positive Symptoms (Andreasen, 1984); HAM-D = Hamilton Depression Rating Scale (Hamilton, 1960); Bunney–Hamburg Scale (Bunney & Hamburg, 1963); PSG = Polysomnography (see Tables 1a for definition); BPRS = Brief Psychiatric Rating Scale (Overall & Gorham, 1962); PANSS = Positive and Negative Symptom Scale (Kay et al., 1987).
